# Hypoxia-Inducible Ubiquitin Specific Peptidase 13 Contributes to Tumor Growth and Metastasis via Enhancing the Toll-Like Receptor 4/Myeloid Differentiation Primary Response Gene 88/Nuclear Factor-κB Pathway in Hepatocellular Carcinoma

**DOI:** 10.3389/fcell.2020.587389

**Published:** 2020-10-19

**Authors:** Shan Gao, Tianxiang Chen, Lijie Li, Xin Liu, Yang Liu, Junjun Zhao, Qiliang Lu, Zhi Zeng, Qiuran Xu, Dongsheng Huang, Kangsheng Tu

**Affiliations:** ^1^Key Laboratory of Tumor Molecular Diagnosis and Individualized Medicine of Zhejiang Province, Zhejiang Provincial People’s Hospital (People’s Hospital of Hangzhou Medical College), Hangzhou, China; ^2^Department of Hepatobiliary Surgery, The First Affiliated Hospital of Xi’an Jiaotong University, Xi’an, China; ^3^The Second Clinical Medical College, Zhejiang Chinese Medical University, Hangzhou, China; ^4^The Medical College of Qingdao University, Qingdao, China; ^5^Graduate Department, Bengbu Medical College, Bengbu, China

**Keywords:** hepatocellular carcinoma, deubiquitinase, ubiquitin-specific protease 13, toll-like receptor 4/myeloid differentiation primary response gene 88/nuclear factor-κB pathway, tumor progression

## Abstract

Hepatocellular carcinoma (HCC) is one of the leading causes of cancer death worldwide. The activation of the toll-like receptor 4/myeloid differentiation primary response gene 88/nuclear factor-κB (TLR4/MyD88/NF-κB) pathway contributes to the development and progression of HCC. The ubiquitin–proteasome system regulates TLR4 expression. However, whether ubiquitin specific peptidase 13 (USP13) stabilizes TLR4 and facilitates HCC progression remains unclear. Here, quantitative real-time PCR (qRT-PCR) and immunohistochemistry analysis revealed that USP13 expression in HCC tissues was higher than in non-tumor liver tissues. Moreover, the elevated expression of USP13 was detected in HCC cells (SK-HEP-1, HepG2, Huh7, and Hep3B) compared to LO2 cells. Interestingly, the positive staining of USP13 was closely correlated with tumor size ≥ 5 cm and advanced tumor stage and conferred to significantly lower survival of HCC patients. Next, USP13 knockdown prominently reduced the proliferation, epithelial–mesenchymal transition (EMT), migration, and invasion of Hep3B and Huh7 cells, while USP13 overexpression enhanced these biological behaviors of HepG2 and LO2 cells. The silencing of USP13 significantly restrained the growth and lung metastasis of HCC cells *in vivo*. Mechanistically, the USP13 depletion markedly inhibited the TLR4/MyD88/NF-κB pathway in HCC cells. USP13 interacted with TLR4 and inhibited the ubiquitin-mediated degradation of TLR4. Significantly, TLR4 re-expression remarkably reversed the effects of USP13 knockdown on HCC cells. USP13 expression was markedly upregulated in HCC cells under hypoxia conditions. Notably, USP13 knockdown repressed hypoxia-induced activation of the TLR4/MyD88/NF-κB pathway in HCC cells. In conclusion, our study uncovered that hypoxia-induced USP13 facilitated HCC progression via enhancing TLR4 deubiquitination and subsequently activating the TLR4/MyD88/NF-κB pathway.

## Introduction

Hepatocellular carcinoma is a dominating histological subtype (about 90%) of primary liver cancer and the second most prevalent cause of cancer-related deaths in China (Chen et al., 2016; Forner et al., 2018). HCC commonly occurs under the conditions of hepatitis B/C (HBV/HCV)-mediated chronic inflammation and metabolic syndrome (Han et al., 2020; McGlynn et al., 2020). Currently, only one third of cases are eligible for curative treatments, and the therapeutic strategies for advanced HCC are limited, which leads to the poor prognosis of patients (Gunasekaran et al., 2020). Therefore, there is an urgent need to thoroughly understand the molecular mechanisms involved in the occurrence and progression of HCC.

Toll-like receptor 4, a well-characterized transmembrane protein, is a crucial sensor involved in inflammation and carcinogenesis (Chen et al., 2008; Yang et al., 2019). The TLR4/myeloid differentiation factor 88 (MyD88) pathway has been recognized as oncogenic signaling in human cancers and is correlated with patients’ poor survival (Chen et al., 2008; Lupi et al., 2020). It has been shown that HCC promotion depends on TLR4 and that TLR4 is required to mediate cell proliferation (Dapito et al., 2012; Weber et al., 2016). The TLR4/MyD88 pathway is activated by HBV infection and induces immune responses against it (Wu et al., 2007). Furthermore, the TLR4/MyD88 pathway plays a vital role in the trilogy of hepatitis–cirrhosis–HCC (Zare-Bidaki et al., 2014; Kang et al., 2018; Song et al., 2018). The inactivation of the TLR4/MyD88 pathway by geniposide reduced signal transducer and activator of transcription 3 (STAT3)/Sp1-dependent vascular endothelial growth factor (VEGF) production in HCC cells (Zhang et al., 2020). A previous study has demonstrated that Baishouwu extract suppresses the initiation and progression of HCC via inhibiting the TLR4/MyD88/NF-κB pathway (Ding et al., 2019). Moreover, hypoxia-inducible factor-1α (HIF-1α)-mediated TLR4/MyD88 pathway participates in hypoxia-induced HCC cell proliferation, migration, and invasion (Zhang et al., 2016). According to the RNA sequencing data from The Cancer Genome Atlas (TCGA) database, we previously found that TLR4 mRNA expression was lower in HCC than that in normal liver tissues. However, immunohistochemistry (IHC) analysis shows that TLR4 is positively expressed in 56.7% of HCC cases (Eiro et al., 2014). These data suggest that TLR4 is regulated by post-translational modifications, such as ubiquitylation and deubiquitylation. Heat shock protein 27 (HSP27) has been identified as a regulator in facilitating endocytosis, ubiquitination, and degradation of TLR4 (Li et al., 2019). US7 and US8, which are encoded by human cytomegalovirus (HCMV), contribute to ubiquitination-mediated degradation of TLR3 and TLR4 (Park et al., 2019). Also, the ubiquitin E3 ligase Triad3A is previously demonstrated to promote TLR4 and TLR9 ubiquitination and degradation (Chuang and Ulevitch, 2004; Lu et al., 2020). Ubiquitin specific protease 13 (USP13), which belongs to the deubiquitinating enzymes (DUBs) superfamily, is implicated in the occurrence and progression of human cancer via promoting deubiquitination and stabilization of substrate proteins, including myeloid cell leukemia 1 (MCL1) (Zhang et al., 2018), phosphatase and tensin homolog (PTEN) (Zhang et al., 2013), melanocyte inducing transcription factor (MITF) (Zhao et al., 2011), Myc (Fang et al., 2017), USP10 (Liu et al., 2011), and RAP80 (Li et al., 2017). A recent study presents that USP13 silencing represses HCC growth possibly by decreasing Myc expression (Huang et al., 2020). Nevertheless, whether USP13 contributes to HCC progression by regulating the toll-like receptor 4/myeloid differentiation primary response gene 88/nuclear factor-κB (TLR4/MyD88/NF-κB) pathway remains unclear.

In this study, we explored the clinical significance of USP13 in HCC and investigated the biological role of USP13 in tumor growth and metastasis *in vitro* and *in vivo*. We also identified the underlying mechanism whereby USP13 promoted the progression of HCC and revealed the regulatory effect of hypoxia on UPS13 expression. Our data indicated that hypoxia-induced USP13 facilitated the proliferation, migration, and invasion of HCC cells via enhancing the TLR4/MyD88/NF-κB pathway.

## Materials and Methods

### Clinical Samples

Hepatocellular carcinoma tissues and corresponding tumor-adjacent tissues were harvested from 80 patients who received hepatectomy at The First Affiliated Hospital of Xi’an Jiaotong University after signing written informed consent. The enrolled participants were pathologically diagnosed with HCC and did not receive preoperative treatment. The tissue samples were obtained and maintained at −80°C for subsequent experiments. The follow-up time was defined from the date of surgical resection to the date of patient death or the last follow-up. The clinicopathologic characters of HCC patients are presented in Table 1.

**TABLE 1:** Correlation between USP13 expression and clinicopathologic characteristics of patients with hepatocellular carcinoma.

**Characteristics**		***n* = 80**	**USP13 expression**		***P-*value**
			**Negative (*n* = 28)**	**Positive (*n* = 52)**	
Age (years)	<50	35	12	23	0.906
	≥50	45	16	29	
Sex	Male	63	21	42	0.547
	Female	17	7	10	
HBV	No	28	12	16	0.280
	Yes	52	16	36	
Serum AFP level (ng/ml)	<20	27	13	14	0.078
	≥20	53	15	38	
Tumor size (cm)	<5	26	15	11	0.003*
	≥5	54	13	41	
No. of tumor nodules	1	65	25	40	0.177
	≥2	15	3	12	
Cirrhosis	No	35	16	19	0.076
	Yes	45	12	33	
Venous infiltration	No	44	18	26	0.221
	Yes	36	10	26	
Edmondson–Steiner grade	I + II	57	21	36	0.587
	III + IV	23	7	16	
TNM stage	I + II	63	26	37	0.024*
	III + IV	17	2	15	

HBV, hepatitis B virus; AFP, alpha-fetoprotein; TNM, tumor-node-metastasis. *Statistically significant.

### Cell Culture and Transfection

Human HCC cell lines (SK-HEP-1, HepG2, Huh7, and Hep3B) and the normal human hepatic cell line (LO2) were obtained from the Cell Bank of Type Culture Collection of the Chinese Academy of Sciences (Shanghai, China) and were maintained in DMEM medium (Gibco, Grand Island, NY, United States) supplemented with 10% fetal bovine serum (FBS, Gibco), 100 μg/ml streptomycin, and 100 U/ml penicillin (Invitrogen, CA, United States) at 37°C in a humidified 5% CO_2_ environment. HCC cells were cultured in a hypoxic incubator (1% O_2_) or treated with cobalt chloride (CoCl_2_, 150 μM, Sigma-Aldrich, St. Louis, MO, United States) to mimic the hypoxia conditions.

The pcDNA3.1-USP13 and pcDNA3.1-TLR4 were generated by inserting the cDNA products of USP13 and TLR4 into the pcDNA3.1 vector (Invitrogen). Plasmids were delivered into HCC cells using Lipofectamine 2000 (Invitrogen) according to the manufacturer’s instruction. Lentivirus-mediated USP13 shRNAs (shUSP13-1 and shUSP13-2) and non-targeting shRNA (NT shRNA) were purchased from GenePharma (Shanghai, China). HCC cells were treated with cycloheximide (CHX, 40 mg/ml, Sigma-Aldrich) and the proteasome inhibitor MG132 (25 μM, Sigma-Aldrich) for protein degradation assay.

### Quantitative Real-Time PCR

Total RNA was isolated from tissues and cells using TRIzol reagent (Invitrogen) and was reversely transcribed into cDNA with a TIANScript RT Kit (Tiangen Biotech, Beijing, China). Amplification and quantification were carried out with a CFX96 Touch^TM^ real-time PCR detection system (Bio-Rad Laboratories, Hercules, CA, United States) using SYBR Green PCR Master Mix (Takara, Shiga, Japan). The relative expression of mRNAs was normalized to GAPDH using 2^–Δ^ ^Δ^ ^*Ct*^ method. The following primers were used: USP13 forward primer: 5′-GCGAAATCAGGCTATTCAGG-3′, reverse primer: 5′-TTGTAAATCACCCATCTTCCTTCC-3′; β-actin forward primer: 5′-GCTTCTCCTTAATGTCACGC-3′; reverse primer: 5′-CCCACACTGTGCCCATCTAC-3′.

### Cell Proliferation Assay

Cell proliferation was determined by Cell Counting Kit-8 (CCK-8) and 5-ethynyl-2′-deoxyuridine (EdU) assay using the commercial CCK-8 kit (Dojindo Laboratories, Dojindo, Japan) and the Cell-Light^TM^ EdU Apollo^®^488 *In Vitro* Imaging Kit (RIBOBIO, Guangzhou, China) as previously described (Wang et al., 2019).

### Transwell Migration and Invasion Assays

Twenty-four hours after transfection, HCC cells were seeded at a density of 1 × 10^4^ cells per well in the upper chamber of transwell insert (8 μm, Corning, Corning, NY, United States). Each upper chamber was covered with or without Matrigel (BD Biosciences, Franklin Lakes, NJ, United States). Moreover, each of the lower chambers contained 500 μl of medium with 10% FBS. The migrated/invaded cells were stained with 0.1% crystal violet and photographed.

### Immunohistochemistry

Immunohistochemistry analysis was performed as previously described (Dou et al., 2019b). The primary USP13 antibody (ab99421) and Ki-67 antibody (ab15580) were obtained from Abcam (Cambridge, MA, United States). Not detected and low staining of USP13 were recognized as negative expression, and medium and high staining of USP13 were defined as positive expression. The percentage of positive staining cells was calculated for quantifying Ki-67 staining.

### Western Blot

The proteins were collected with lysis buffer (Beyotime, Shanghai, China) containing protease and phosphatase inhibitor (Thermo Fisher Scientific, Waltham, MA, United States) and quantified using Pierce BCA Protein Assay Kit (Thermo Fisher Scientific). Protein samples were separated using 6%–10% sodium dodecyl sulfate-polyacrylamide gels and transferred onto polyvinylidene difluoride (PVDF) membranes (Millipore, Bedford, MA, United States). After blocking with 5% non-fat milk, the membranes were incubated overnight at 4°C with primary antibodies against USP13 (ab99421, Abcam), TLR4 (sc-293072, Santa Cruz Biotechnology, Dallas, TX, United States), MyD88 (#50010, Cell Signaling Technology, Beverly, MA, United States), ubiquitin (Ub, ab7780, Abcam), phospho-NF-κB p65 (Ser536) (#3033, Cell Signaling Technology), E-cadherin (20874-1-AP, Proteintech, Wuhan, China), N-cadherin (22018-1-AP, Proteintech), vimentin (10366-1-AP, Proteintech), and HIF-1α (10006421, Cayman Chemical, Ann Arbor, MI, United States). Anti-β-actin antibody (ab8227, Abcam) was used as an internal control. The next day, the membranes were probed with horseradish peroxidase (HRP)-conjugated secondary antibody (Beyotime) for 1 h at room temperature. Finally, blots were developed using the ECL kit (Millipore, United States) and semi-quantified by ImageJ software (1.46; National Institutes of Health, Bethesda, MD, United States).

### Co-immunoprecipitation Assay

The specific antibody against TLR4 (sc-293072, Santa Cruz Biotechnology) was used for the co-immunoprecipitation (co-IP) assay, which was performed according to the previously described protocols (Tu et al., 2014).

### *In vivo* Experiments

Four-week BALB/c nude mice were purchased from Shanghai SLAC Laboratory Animal Company (Shanghai, China). The *in vivo* tumor growth and lung metastasis experiments were performed using Hep3B cells with or without USP13 knockdown according to the protocols as previously described (Dou et al., 2019a; Guo et al., 2019). The tumor volume (*V*) was calculated as follows: *V* = (*L* × *D*2)/2 where *L* and *D* represent the tumor length and width, respectively (in mm). Three weeks after subcutaneous injection, the mice were euthanized, and tumor tissues were harvested for IHC staining of USP13 and Ki-67. Eight weeks after tail vein injection, the mice were sacrificed under euthanization, and lung tissues were collected for hematoxylin and eosin (H&E) staining. The animal study was approved by the Institutional Animal Care and Use Committee of Xi’an Jiaotong University.

### Microarray

Hep3B cells were cultured under normoxic and hypoxic conditions, respectively, for 48 h. Then, total RNA was isolated from Hep3B cells using TRIzol reagent (Invitrogen). RNA quantity and quality were measured by NanoDrop ND-1000 (Thermo Scientific). The Arraystar Human LncRNA Arrays V5 (Arraystar, Rockville, MD, United States) was used to identify differently expressed lncRNA and mRNA based on the manufacturer’s standard protocols (Aksomics, Shanghai, China). Arraystar Human LncRNA Microarray V5.0 is designed for the global profiling of human LncRNAs and protein-coding transcripts. This third-generation LncRNA microarray can detect about 39,317 LncRNAs and 21,174 coding transcripts. The microarray data (GSE155505) was uploaded into the Gene Expression Omnibus (GEO).

### Statistical Analysis

The date of three independent experiments is shown as mean ± standard deviation (SD). Comparison among two groups or >2 groups was executed with one-way analysis of variance (ANOVA) or Student’s *t*-test. The survival of HCC patients was analyzed using the Kaplan–Meier’s method and log-rank test. Chi-square test was employed to explore the correlations between clinical variables and USP13 expression. All statistical analyses were carried out with GraphPad Prism 8.0 (GraphPad Inc., San Diego, CA, United States). *P* < 0.05 was defined to be statistically significant.

## Results

### USP13 Is Highly Expressed in HCC

**FIGURE 1:**
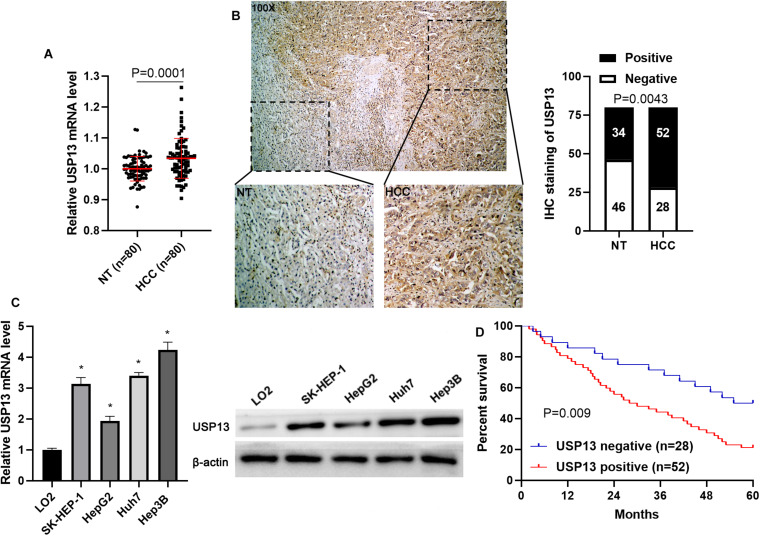
USP13 expression is upregulated in HCC. **(A)** qRT-PCR was performed to detect USP13 mRNA levels in 80 pairs of HCC and adjacent non-tumor (NT) tissues. **(B)** IHC staining of USP13 in a representative sample containing HCC and adjacent NT tissues. The staining intensity of USP13 in HCC was significantly higher than that in NT tissues. **(C)** The expression of USP13 protein in HCC cell lines (SK-HEP-1, HepG2, Huh7, and Hep3B) and a normal hepatic cell line (LO2). **(D)** HCC patients with positive expression of USP13 showed an apparent shorter overall survival than cases with negative expression of USP13. *P < 0.05.

To determine the expression difference of USP13 between HCC and tumor-adjacent tissues, qRT-PCR and IHC analysis were performed to detect USP13 mRNA and protein levels, respectively. We found that UPS13 mRNA expression in HCC tissues was higher than that in non-tumor liver tissues (*P* = 0.0001, Figure 1A). TCGA data analysis using GEPIA web tool (Tang et al., 2017) consistently revealed a significantly higher level of USP13 mRNA in HCC (*P* < 0.0001, Supplementary Figure S1A). Furthermore, IHC analysis indicated that USP13 positive expression was confirmed in 52 of 80 (65.0%) HCC cases, while only 34 of 80 (42.5%) non-tumor samples showed USP13 positive expression (*P* = 0.0043, Figure 1B). Besides, the level of USP13 in HCC cell lines (SK-HEP-1, HepG2, Huh7, and Hep3B) was prominently higher than that in LO2 cells (*P* < 0.05, Figure 1C). These data indicated an oncogenic role of USP13 in HCC.

### The Positive Expression of USP13 Correlates With Poor Prognosis of HCC

Next, the clinical significance of USP13 in HCC was determined in this study. As shown in Table 1, the positive expression of USP13 was significantly associated with tumor size ≥ 5 cm (*P* = 0.003) and advanced tumor-node-metastasis (TNM) stage (III + IV, *P* = 0.024), as analyzed by chi-square test. Moreover, HCC patients with USP13 positive expression had a significantly lower overall survival compared to cases with USP13 negative expression (*P* = 0.009, Figure 1D). More importantly, TCGA data analysis using the GEPIA web tool (Tang et al., 2017) also indicated that the high USP13 mRNA level indicated an apparent shorter overall survival and disease-free survival of HCC patients (*P* = 0.0026 and 0.058, respectively, Supplementary Figure S1B). Thus, our results suggested that USP13 might be a promising prognostic biomarker for HCC.

### USP13 Knockdown Suppresses HCC Cell Proliferation and Invasion *in vitro* and *in vivo*

**FIGURE 2:**
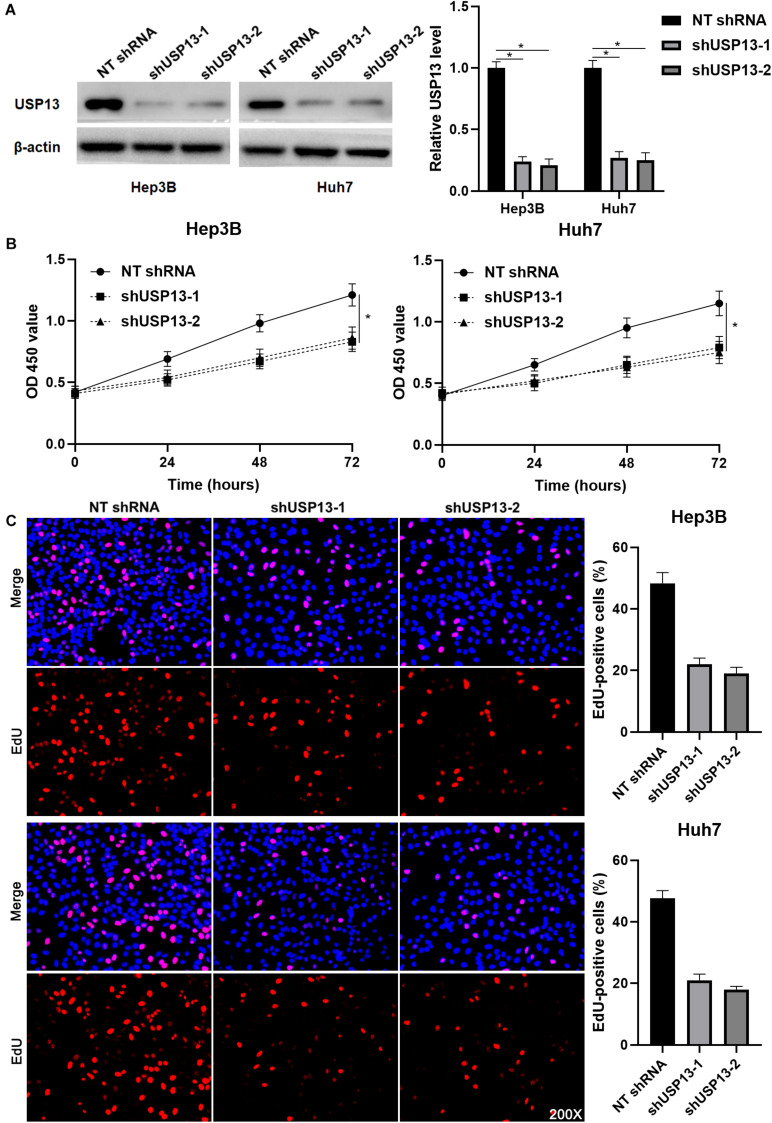
USP13 knockdown inhibits the proliferation of HCC cells. **(A)** Hep3B and Huh7 cells were transfected with non-targeting (NT) shRNA and USP13 shRNAs (shUSP13-1 and shUSP13-2), respectively, and detected by western blot (WB) for USP13 expression. **(B)** CCK-8 assay indicated that USP13 silencing significantly reduced the viability of HCC cells. **(C)** The silencing of USP13 repressed the proliferation of HCC cells as suggested by EdU assay. *P < 0.05.

**FIGURE 3:**
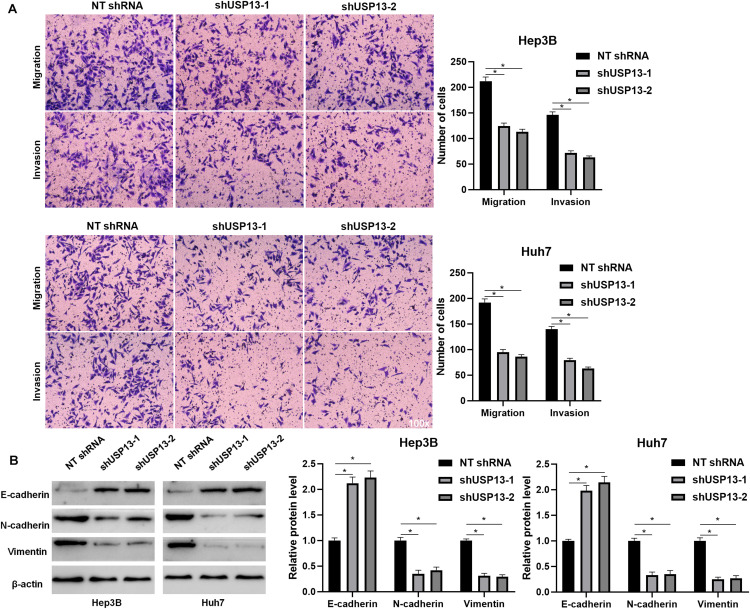
USP13 depletion suppressed the EMT, migration, and invasion of HCC cells. **(A)** USP13 knockdown by two different shRNAs significantly reduced the number of migrated and invaded HCC cells. **(B)** Western blotting analysis indicated that USP13 knockdown increased E-cadherin expression and reduced N-cadherin and vimentin levels in HCC cells. *P < 0.05.

**FIGURE 4:**
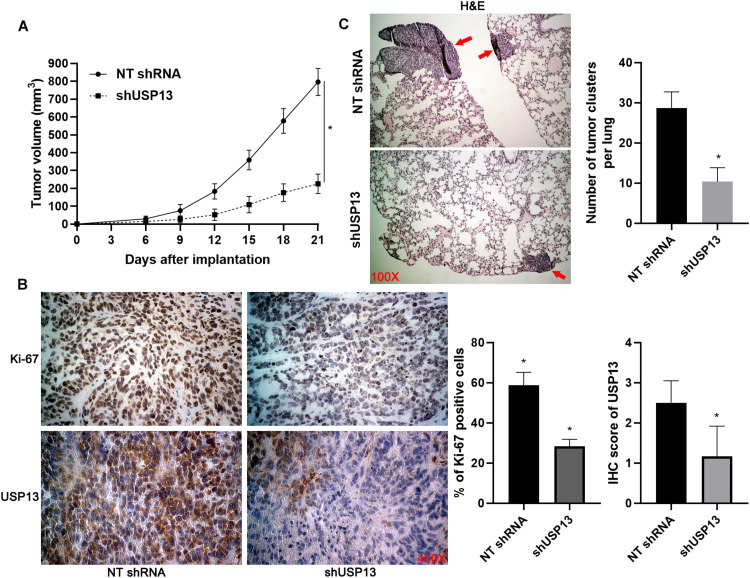
USP13 knockdown inhibits HCC growth and metastasis in mice. **(A)** Hep3B cells with or without USP13 knockdown were subcutaneously injected into nude mice (*n* = 6 for each group). Tumor growth curves indicated that USP13 knockdown restrained HCC growth *in vivo*. **(B)** IHC staining of Ki-67 and USP13 was performed in subcutaneous tumor tissues. **(C)** Hep3B cells with or without USP13 knockdown were injected into nude mice via tail vein (*n* = 6 for each group). H&E staining of lung tissues suggested that USP13 knockdown reduced lung metastasis of HCC in mice. *P < 0.05.

Hep3B and Huh7 cells, which expressed a relatively higher level of USP13, were transfected with two independent shRNAs to specifically downregulate USP13 (*P* < 0.05, Figure 2A). Both CCK-8 and EdU assays consistently indicated that USP3 knockdown markedly reduced the proliferation of HCC cells (*P* < 0.05, Figures 2B,C). Furthermore, the silencing of USP13 remarkably repressed HCC cell migration and invasion as determined by transwell assay (*P* < 0.05, Figure 3A). Western blotting data indicated that USP13 knockdown increased E-cadherin expression and reduced N-cadherin and vimentin levels in HCC cells (*P* < 0.05, Figure 3B). Conversely, USP13 overexpression significantly enhanced the proliferation, epithelial–mesenchymal transition (EMT), migration, and invasion of HepG2 and LO2 cells (*P* < 0.05, Supplementary Figures S2, S3). Then, the effects of USP13 knockdown on HCC cells were further confirmed *in vivo*. The growth curves of subcutaneous tumors formed by Hep3B cells suggested that USP13 silencing inhibited tumor growth in mice (*P* < 0.05, Figure 4A). Furthermore, tumor tissues from the USP13 knockdown group had a prominently lower USP13 and Ki-67 staining density compared to samples from the control group (*P* < 0.05, Figure 4B). H&E staining of lung tissues collected from the mouse HCC metastasis model indicated that the depletion of USP13 markedly reduced the number of lung metastases *in vivo* (*P* < 0.05, Figure 4C). Collectively, our data identified USP13 as an oncogene in HCC.

### USP13 Regulates TLR4/MyD88/NF-κB Pathway via Enhancing TLR4 Stabilization

**FIGURE 5:**
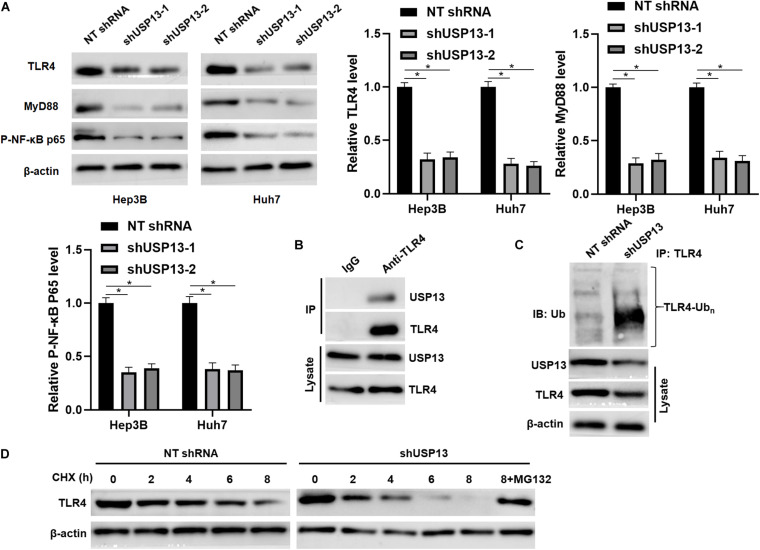
USP13 knockdown reduced the stabilization of TLR4. **(A)** Hep3B and Huh7 cells were transfected with non-targeting (NT) shRNA and USP13 shRNAs (shUSP13-1 and shUSP13-2), respectively, and detected by WB for TLR4, MyD88, and P-NF-κB p65 expression. **(B)** USP13 was immunoprecipitated by TLR4 in Hep3B cells. **(C)** USP13 silencing increased the ubiquitination of TLR4 in Hep3B cells. **(D)** Hep3B cells, with or without USP13 knockdown, were treated with CHX (40 mg/ml) to block protein synthesis. WB was performed to detect TLR4 level at different time points. The proteasome inhibitor MG132 (25 μM) was used to suppress TLR4 degradation. *P < 0.05.

Since TLR4 stabilization is regulated by ubiquitination-mediated degradation (Chuang and Ulevitch, 2004; Lu et al., 2020), TCGA data analysis using the GEPIA web tool (Tang et al., 2017) indicated that the expression of TLR4 mRNA in HCC tissues was significantly lower than that in normal liver tissues (*P* < 0.0001, Supplementary Figure S4A). We aimed to know whether deubiquitinase USP13 participated in regulating TLR4 stabilization in HCC. Interestingly, we found that USP13 knockdown significantly reduced the level of TLR4 protein in Hep3B and Huh7 cells (*P* < 0.05, Figure 5A). The co-IP assay revealed that USP13 directly interacted with TLR4 in Hep3B cells (Figure 5B). Notably, USP13 knockdown increased the ubiquitination of TLR4 in Hep3B cells (Figure 5C). Hep3B cells were treated with CHX to block new protein synthesis. Then, we found that TLR4 protein was degraded faster in the USP13 group than in the control group (Figure 5D). However, proteasome inhibitor MG132 treatment could reduce the degradation of TLR4 induced by USP13 knockdown (Figure 5D). These results indicated that USP13 increased TLR4 abundance via enhancing the deubiquitination of TLR4 in HCC cells. Next, we determined the effects of USP13 knockdown on the TLR4/MyD88/NF-κB pathway in HCC cells. As expected, USP13 knockdown markedly reduced the levels of MyD88 and P-NF-κB p65 in HCC cells (*P* < 0.05, Figure 5A). The levels of TLR4 and MyD88 protein in HCC cells were markedly higher than those in LO2 cells (*P* < 0.05, Supplementary Figure S4B). Moreover, the expressions of TLR4, MyD88, and P-NF-κB p65 in subcutaneous tumor tissues from the USP13 knockdown group were prominently lower than those in the control group (*P* < 0.05, Supplementary Figure S4C).

### TLR4 Re-expression Reverses the Effects of USP13 Knockdown on HCC Cells

**FIGURE 6:**
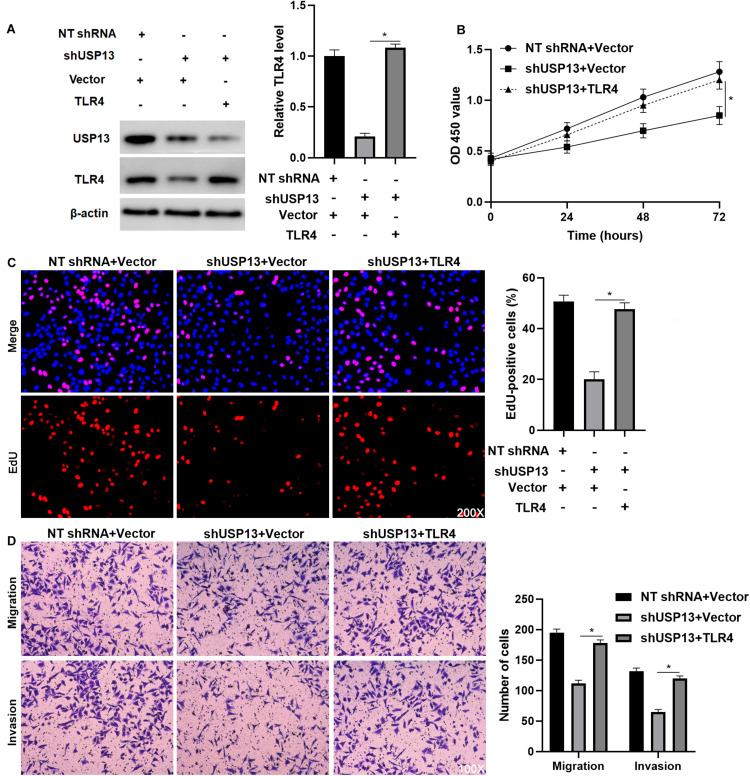
TLR4 re-expression abrogates the effects of USP13 knockdown on Hep3B cells. **(A)** TLR4 expression was rescued by plasmid transfection in Hep3B cells with USP13 knockdown. **(B)** CCK-8, **(C)** EdU, and **(D)** transwell assays were performed to assess the proliferation, migration, and invasion of Hep3B cells after transfecting with corresponding vectors. *P < 0.05.

To further investigate whether TLR4 mediated the oncogenic role of USP13 in HCC, TLR4 was re-expressed via plasmid transfection in Hep3B cells with USP13 knockdown (*P* < 0.05, Figure 6A). As shown in Figures 6B,C, TLR4 restoration significantly enhanced USP13 knockdown Hep3B cells’ proliferation (*P* < 0.05). Furthermore, TLR4 re-expression reversed USP13 knockdown-induced inhibitory effects on Hep3B cell migration and invasion (*P* < 0.05, Figure 6D). Similarly, TLR4 markedly abolished the effects of USP13 knockdown on Huh7 cell proliferation, migration, and invasion (*P* < 0.05, Supplementary Figure S5). Altogether, our results demonstrated that USP13 promoted HCC progression via regulating the TLR4/MyD88/NF-κB pathway.

### Hypoxia Activates the TLR4/MyD88/NF-κB Pathway via Inducing USP13 in HCC Cells

**FIGURE 7:**
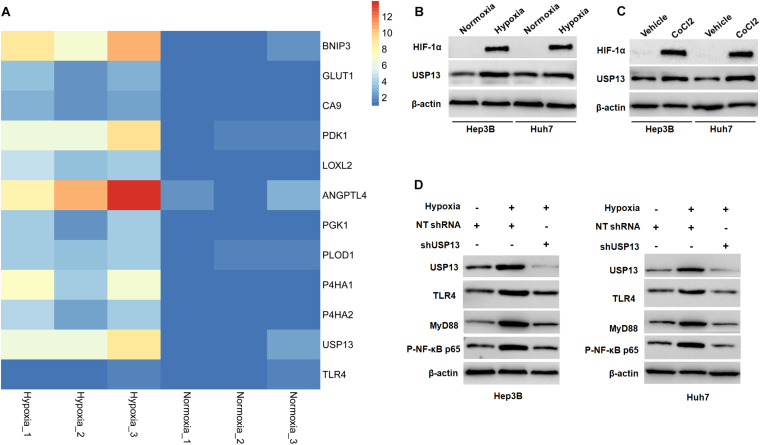
Hypoxia activates the USP13/TLR4/MyD88/NF-κB pathway in HCC cells. **(A)** Heatmap of the differentially expressed genes, including USP13, TLR4, and 10 HIF target genes. **(B)** The levels of HIF-1α and USP13 protein were increased in Hep3B and Huh7 cells under hypoxia conditions. **(C)** CoCl_2_ treatment (150 μM) resulted in the increased levels of HIF-1α and USP13 protein in Hep3B and Huh7 cells. **(D)** Hypoxia increased USP13, TLR4, MyD88, and P-NF-κB p65 proteins, which was subsequently reduced by USP13 knockdown.

Since hypoxia has been recognized as an inducer of the activation of the TLR4/MyD88/NF-κB pathway in HCC (Won et al., 2015; Zhang et al., 2016), therefore, we aimed to investigate whether USP13 mediated the hypoxia-induced activation of the TLR4/MyD88/NF-κB pathway in HCC. Our microarray data indicated that hypoxia increased the expression of 10 HIF target-gene mRNAs and resulted in significantly increased expression of USP13 mRNA in Hep3B cells (Figure 7A), while the expression of TLR4 mRNA was not prominently impacted under hypoxia (Figure 7A). Furthermore, either hypoxia or CoCl_2_ treatment markedly upregulated the levels of HIF-1α and USP13 protein in Hep3B and Huh7 cells (Figures 7B,C). Interestingly, the USP13 knockdown repressed the hypoxia-induced activation of the TLR4/MyD88/NF-κB pathway in HCC cells (Figure 7D). Altogether, our results suggested that USP13 played an essential role in the hypoxia-mediated TLR4/MyD88/NF-κB pathway in HCC.

## Discussion

Ubiquitination-mediated protein degradation participates in several physiological and pathological processes, including cancer (Popovic et al., 2014; Xu et al., 2017b). Deubiquitinase USP13 is aberrantly expressed in several human cancers and correlates with poor prognosis (Zhang et al., 2013; Han et al., 2016; Fang et al., 2017; Man et al., 2019). For example, USP13 is overexpressed in ovarian cancer, and its overexpression significantly predicts poor clinical outcomes (Han et al., 2016). USP13 expression is upregulated in glioblastoma (GBM) tissues and inversely correlated with patients’ overall survival (Fang et al., 2017). However, the underexpression of USP13 is observed in breast cancer and bladder cancer (Zhang et al., 2013; Man et al., 2019). Our data and TCGA data consistently revealed the upregulated expression of USP13 in HCC tissues compared to non-tumor liver tissues in the current study. Notably, the positive expression of USP13 was associated with unfavorable clinical features, such as tumor size ≥ 5 cm and advanced TNM stage (III + IV), and indicated poor prognosis of HCC patients. Thus, USP13 overexpression in HCC tissues might be a potential indicator of poor clinical outcomes of patients. The expression of USP13 in HCC cells was significantly higher than that in a normal hepatic cell line. Tumor heterogeneity leads to differential expression of USP13 in different HCC cell lines.

A previous study reports that USP13 knockdown suppresses ovarian cancer cell proliferation *in vitro* and tumor formation *in vivo* (Han et al., 2016). Moreover, USP13 depletion represses the proliferation of glioma stem cells (GSCs) and restrains tumor growth in mice (Fang et al., 2017). In contrast, the loss of USP13 facilitates the proliferation, glycolysis, and anchorage-independent growth of breast cancer cells (Zhang et al., 2013). The bladder cell proliferation, migration, and invasion potentials are enhanced by USP13 knockdown (Man et al., 2019). The upregulated expression of USP13 in tumor tissues indicated that it might function as an oncogenic in HCC. As expected, we found that USP13 knockdown markedly inhibited the proliferation, EMT, migration, and invasion of HCC cells and significantly repressed tumor growth and lung metastasis of HCC *in vivo*. Besides, the ectopic expression of USP13 facilitated the proliferation, EMT, migration, and invasion of HepG2 and LO2 cells *in vitro*.

As a deubiquitinase, USP13 regulates protein abundance via enhancing deubiquitylation and stabilization of the substrate. Several proteins, such as MCL1 (Zhang et al., 2018), PTEN (Zhang et al., 2013), MITF (Zhao et al., 2011), Myc (Fang et al., 2017), USP10 (Liu et al., 2011), and RAP80 (Li et al., 2017), have been demonstrated to be regulated by USP13 for deubiquitylation and stabilization. However, it is still unknown whether USP13 deubiquitinates and thus stabilizes TLR4 in HCC. Here, we demonstrated that USP13 enhanced the stabilization of TLR4 via direct binding and deubiquitylation of TLR4. Accordingly, TLR4 was recognized as a novel substrate of USP13 in HCC. The structural features of TLR4 include an ectodomain consisting of multiple leucine-rich repeats and a cysteine-rich domain, a transmembrane region, and a highly conserved cytoplasmic Toll–interleukin 1 receptor (IL-1R) domain (TIR domain) (Chuang and Ulevitch, 2001). The putative ligand-binding occurs within the ectodomain, whereas the TIR domain provides a key site for interactions with intracellular proteins such as members of the MyD88 adaptor protein family (Aderem and Ulevitch, 2000; Takeda et al., 2003). An E3 ubiquitin-protein ligase Triad3A binds to the cytoplasmic tail (TIR domain) of TLR4 and targets TLR4 for ubiquitination and proteolytic degradation (Chuang and Ulevitch, 2004). In this study, a co-IP assay demonstrated the interaction between USP13 and TLR4 in HCC cells. Since USP13 is an intracellular protein, we suggest that USP13 binds to the TIR domain of TLR4 in HCC cells. TLR4 and its adaptor MyD88 have been reported as oncogenic signaling in several human cancers, including HCC (Apetoh et al., 2007; Rathore et al., 2019; Zhang et al., 2020). Recent studies have shown that the TLR4/MyD88 pathway regulates NF-κB signaling and VEGF, IL-23, and IL-17A expression in HCC (Kang et al., 2018; Ding et al., 2019; Zhang et al., 2020). The inhibition of the TLR4/MyD88 pathway represses the occurrence and progression of HCC (Ding et al., 2019; Zhang et al., 2020). NF-κB-mediated EMT process contributes to HCC progression, and the suppression of the NF-κB pathway represses EMT and invasion of tumor cells (Song et al., 2014; Zhu et al., 2016). Importantly, USP13 knockdown markedly resulted in the inactivation of the TLR4/MyD88/NF-κB pathway in HCC cells. TLR4 re-expression reversed the inhibitory effects of USP13 knockdown on the proliferation, migration, and invasion of HCC cells. Thus, USP13 contributed to HCC progression possibly by targeting the TLR4/MyD88/NF-κB pathway.

Hypoxia, which resulted from rapid tumor growth and vascular abnormality, has been identified as a critical driver of HCC progression (Jing et al., 2019). Our previous studies have demonstrated that hypoxia contributes to the growth and metastasis of HCC via regulating protein-coding genes and non-coding RNAs, such TUFT1 (Dou et al., 2019b), VASP (Liu et al., 2018), miR-1296 (Xu et al., 2017a), and RUNX1-IT1 (Sun et al., 2020). Hypoxia has been recognized as an inducer of the activation of the TLR4/MyD88/NF-κB pathway in hepatic ischemia/reperfusion injury and liver fibrosis (Kang et al., 2017; Du et al., 2019). Moreover, HIF-1α knockdown inactivates the TLR4/MyD88 pathway and abrogates hypoxia-induced proliferation, migration, and invasion of HCC cells (Zhang et al., 2016). Our data showed that hypoxia increased the expression of USP13 mRNA, while it did not impact the level of TLR4 mRNA in Hep3B cells, which further support the post-transcriptional regulation of TLR4 by USP13. Besides, either hypoxia or CoCl_2_ treatment upregulated the level of USP13 protein in HCC cells. Significantly, USP13 knockdown inhibited hypoxia-induced activation of the TLR4/MyD88/NF-κB pathway in HCC cells. These results provide new insight into the underlying mechanism involved in hypoxia-induced TLR4/MyD88/NF-κB pathway activation in HCC.

In summary, our findings elucidated that the upregulated expression of USP13 in HCC tissues conferred to poor clinical outcome. We provided evidence to support that USP13, induced by hypoxia, promoted HCC progression by maintaining the TLR4/MyD88/NF-κB pathway. These data might provide novel insights into the pathogenesis of HCC.

## Data Availability Statement

All datasets presented in this study are included in the article/Supplementary Material.

## Ethics Statement

The studies involving human participants were reviewed and approved by Research Ethics Committee of The First Affiliated Hospital of Xi’an Jiaotong University. The patients/participants provided their written informed consent to participate in this study. The animal study was reviewed and approved by Institutional Animal Care and Use Committee of Xi’an Jiaotong University.

## Author Contributions

QX, DH, and KT conceived and designed the experiments. SG, TC, LL, XL, YL, and JZ performed the experiments. SG, TC, and LL analyzed the data. QL and ZZ contributed reagents, materials, and analysis tools. SG and KT wrote the manuscript. All authors read and approved the final manuscript.

## Conflict of Interest

The authors declare that the research was conducted in the absence of any commercial or financial relationships that could be construed as a potential conflict of interest.

## Supplementary Material

Supplementary Figure 1 The expression and prognostic significance of USP13 in TCGA data. **(A)** TCGA data analysis via the GEPIA web tool indicated that USP13 mRNA expression in HCC was significantly higher than that in normal liver tissues. **(B)** HCC patients with high USP13 level predicted a lower disease-free survival and overall survival.

Supplementary Figure 2 USP13 overexpression promotes the proliferation, EMT, migration, and invasion of HepG2 cells. **(A)** HepG2 cells were transfected with pcDNA3.1-USP13 and empty vector (EV), respectively, and detected by WB for USP13 expression. **(B)** CCK-8 assay indicated that USP13 overexpression significantly increased the viability of HepG2 cells. **(C)** Ectopic expression of USP13 enhanced the proliferation of HepG2 cells as suggested by EdU assay. **(D)** Overexpression of USP13 markedly promoted the migration and invasion of HepG2 cells. **(E)** USP13 overexpression reduced E-cadherin expression but increased the levels of N-cadherin and vimentin in HepG2 cells. ^∗^*P* < 0.05.

Supplementary Figure S3 USP13 overexpression promotes the proliferation, EMT, migration, and invasion of LO2 cells. **(A)** LO2 cells were transfected with pcDNA3.1-USP13 and empty vector (EV), respectively, and detected by WB for USP13 expression. **(B)** CCK-8 assay indicated that USP13 overexpression significantly increased the viability of LO2 cells. **(C)** Ectopic expression of USP13 enhanced the proliferation of LO2 cells, as suggested by EdU assay. **(D)** Overexpression of USP13 markedly promoted the migration and invasion of LO2 cells. **(E)** USP13 overexpression reduced E-cadherin expression but increased the levels of N-cadherin and vimentin in LO2 cells. ^∗^*P* < 0.05.

Supplementary Figure 4 The levels of TLR4, MyD88, and P-NF-κB p65 in HCC tissues and cells. **(A)** TCGA data analysis via the GEPIA web tool indicated that TLR4 mRNA expression in HCC was significantly lower than in that in normal liver tissues. **(B)** The levels of TLR4 and MyD88 protein in HCC cell lines (SK-HEP-1, HepG2, Huh7, and Hep3B) and a normal hepatic cell line (LO2). **(C)** The expressions of TLR4, MyD88, and P-NF-κB p65 in subcutaneous tumor tissues from the USP13 knockdown group were prominently lower than those in the control group. ^∗^*P* < 0.05.

Supplementary Figure 5 TLR4 restoration reverses USP13 knockdown-induced effects on Huh7 cells. **(A)** CCK-8, **(B)** EdU, and **(C)** transwell assays were performed to assess the proliferation, migration, and invasion of Huh7 cells after transfecting with corresponding vectors. ^∗^*P* < 0.05.

## Abbreviations

HCC: hepatocellular carcinoma
MyD88: myeloid differentiation primary response gene 88
NF- κ B: nuclear factor- κ B
TLR4: toll-like receptor 4
USP13: ubiquitin specific peptidase 13.
