# Supplementary figures and images for: Hypoxia-Inducible Ubiquitin Specific Peptidase 13 Contributes to Tumor Growth and Metastasis via Enhancing the Toll-Like Receptor 4/Myeloid Differentiation Primary Response Gene 88/Nuclear Factor-κB Pathway in Hepatocellular Carcinoma
Source: Front Cell Dev Biol. 2020 Oct 19;8:587389. doi: 10.3389/fcell.2020.587389 (PMC7604352; doi:10.3389/fcell.2020.587389)

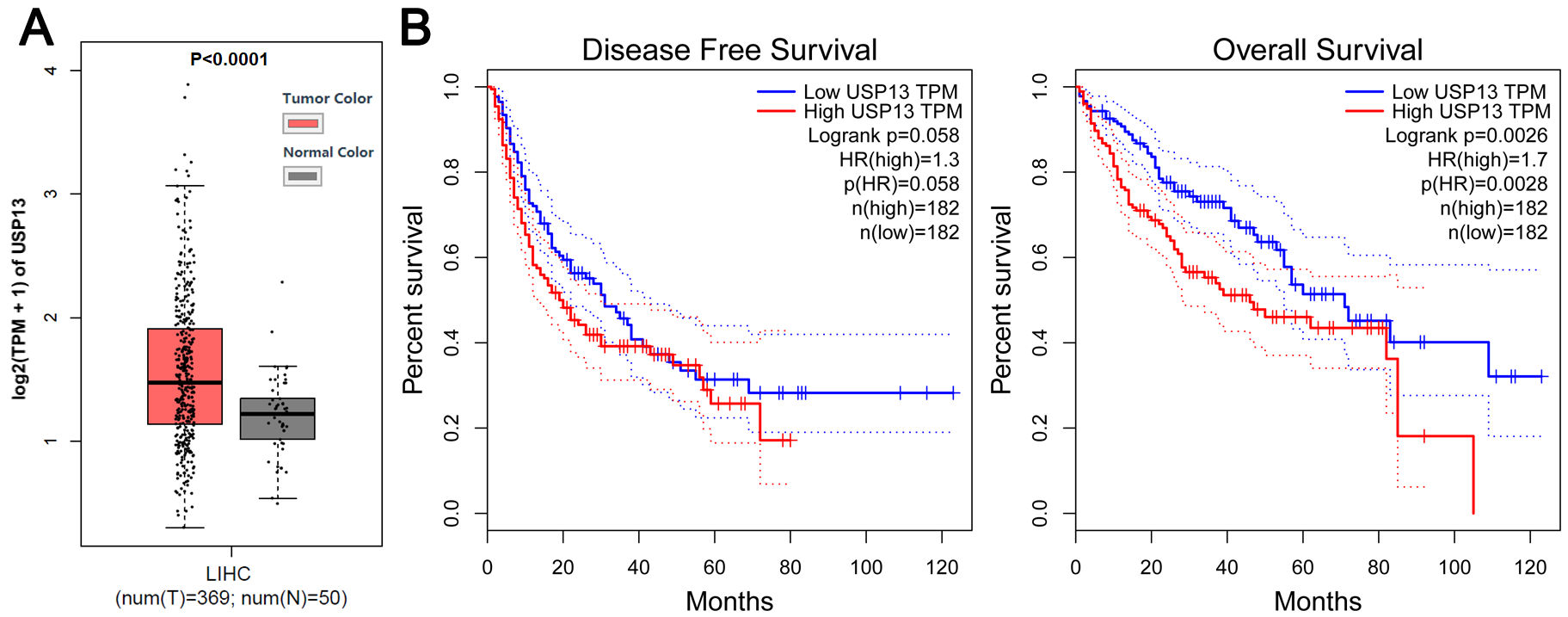

Supplement: Supplementary Figure 1 — The expression and prognostic significance of USP13 in TCGA data. (A) TCGA data analysis via the GEPIA web tool indicated that USP13 mRNA expression in HCC was significantly higher than that in normal liver tissues. (B) HCC patients with high USP13 level predicted a lower disease-free survival and overall survival. [file Image_1.TIF]

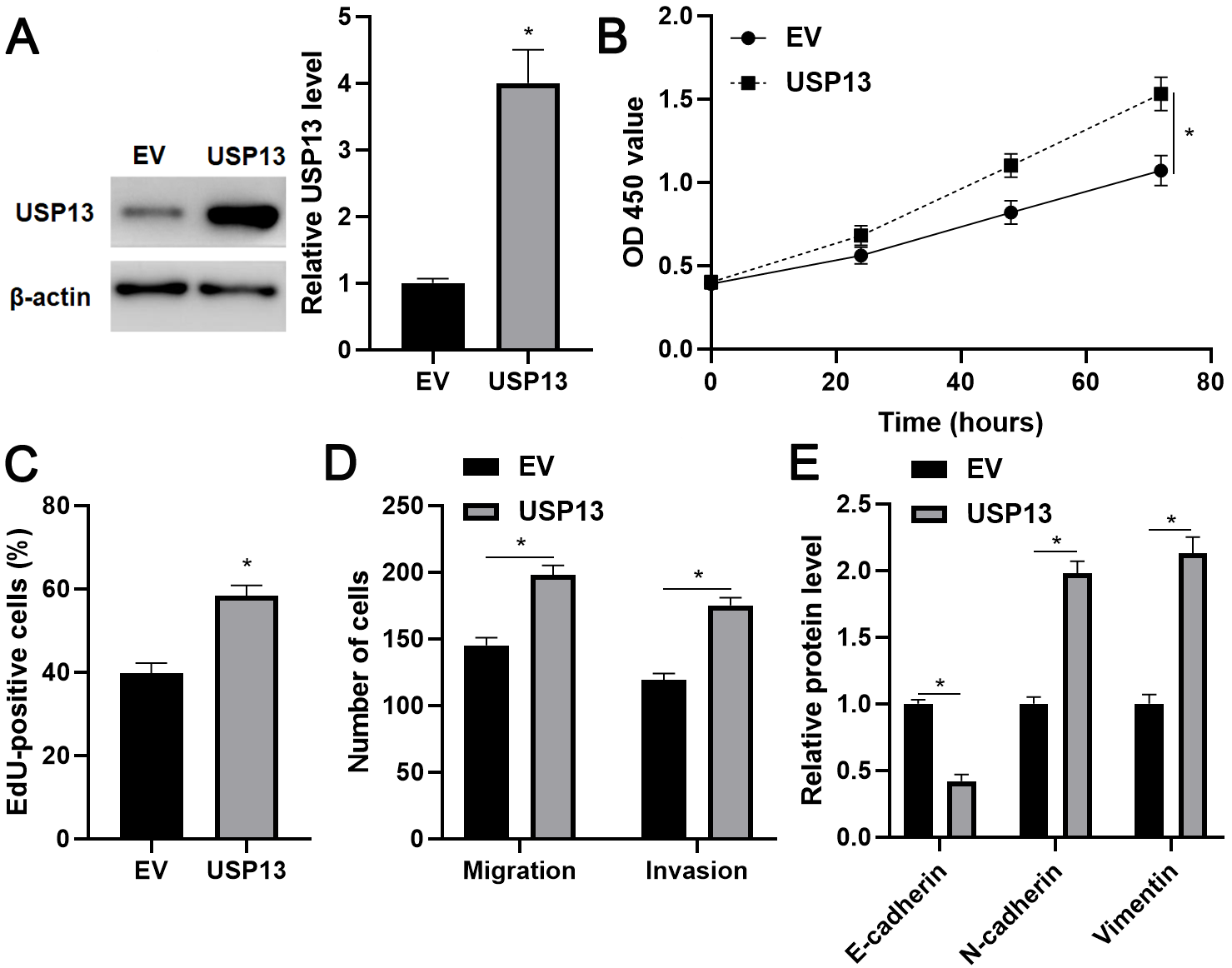

Supplement: Supplementary Figure 2 — USP13 overexpression promotes the proliferation, EMT, migration, and invasion of HepG2 cells. (A) HepG2 cells were transfected with pcDNA3.1-USP13 and empty vector (EV), respectively, and detected by WB for USP13 expression. (B) CCK-8 assay indicated that USP13 overexpression significantly increased the viability of HepG2 cells. (C) Ectopic expression of USP13 enhanced the proliferation of HepG2 cells as suggested by EdU assay. (D) Overexpression of USP13 markedly promoted the migration and invasion of HepG2 cells. (E) USP13 overexpression reduced E-cadherin expression but increased the levels of N-cadherin and vimentin in HepG2 cells. ∗P < 0.05. [file Image_2.TIF]

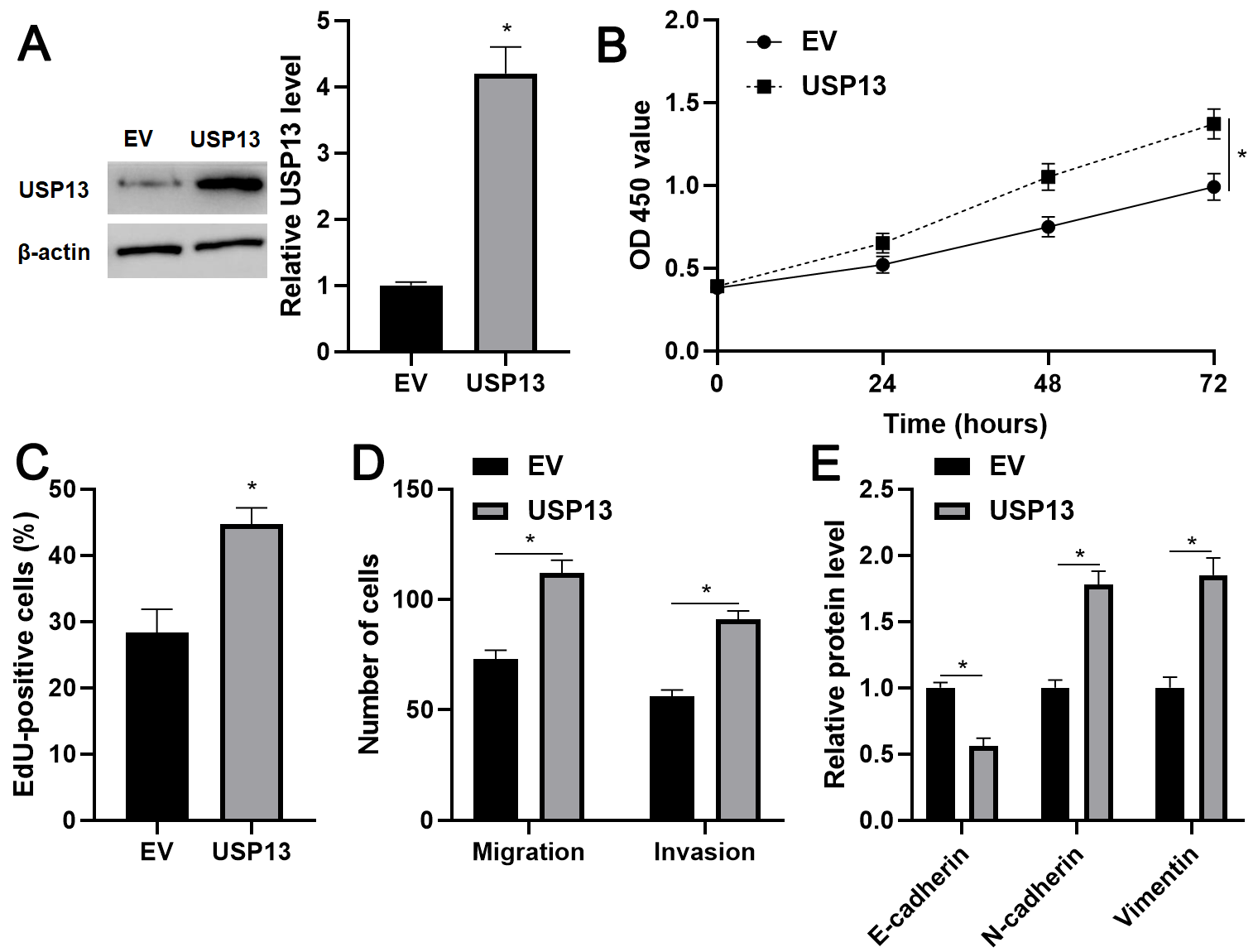

Supplement: Supplementary Figure S3 — USP13 overexpression promotes the proliferation, EMT, migration, and invasion of LO2 cells. (A) LO2 cells were transfected with pcDNA3.1-USP13 and empty vector (EV), respectively, and detected by WB for USP13 expression. (B) CCK-8 assay indicated that USP13 overexpression significantly increased the viability of LO2 cells. (C) Ectopic expression of USP13 enhanced the proliferation of LO2 cells, as suggested by EdU assay. (D) Overexpression of USP13 markedly promoted the migration and invasion of LO2 cells. (E) USP13 overexpression reduced E-cadherin expression but increased the levels of N-cadherin and vimentin in LO2 cells. ∗P < 0.05. [file Image_3.TIF]

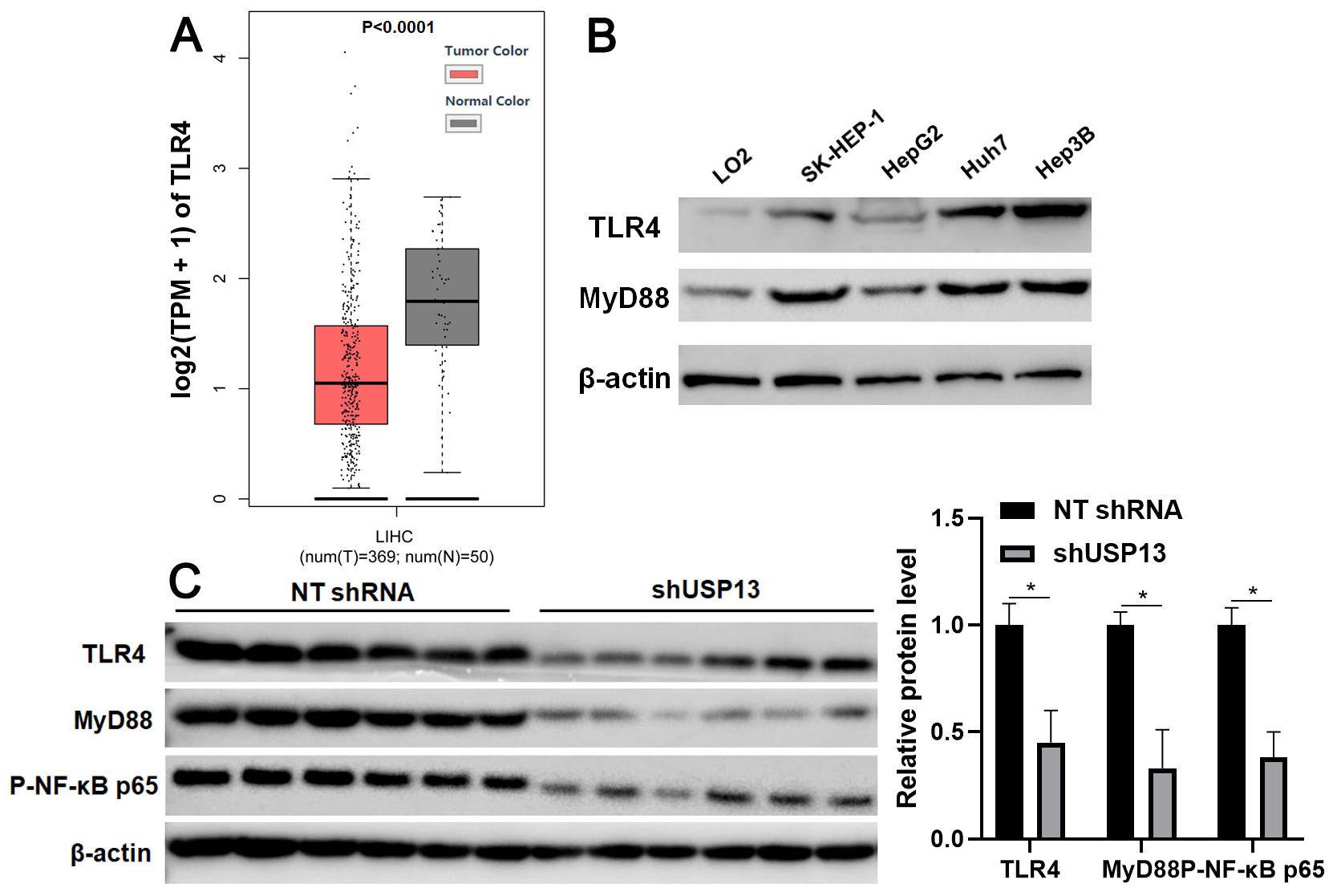

Supplement: Supplementary Figure 4 — The levels of TLR4, MyD88, and P-NF-κB p65 in HCC tissues and cells. (A) TCGA data analysis via the GEPIA web tool indicated that TLR4 mRNA expression in HCC was significantly lower than in that in normal liver tissues. (B) The levels of TLR4 and MyD88 protein in HCC cell lines (SK-HEP-1, HepG2, Huh7, and Hep3B) and a normal hepatic cell line (LO2). (C) The expressions of TLR4, MyD88, and P-NF-κB p65 in subcutaneous tumor tissues from the USP13 knockdown group were prominently lower than those in the control group. ∗P < 0.05. [file Image_4.TIF]

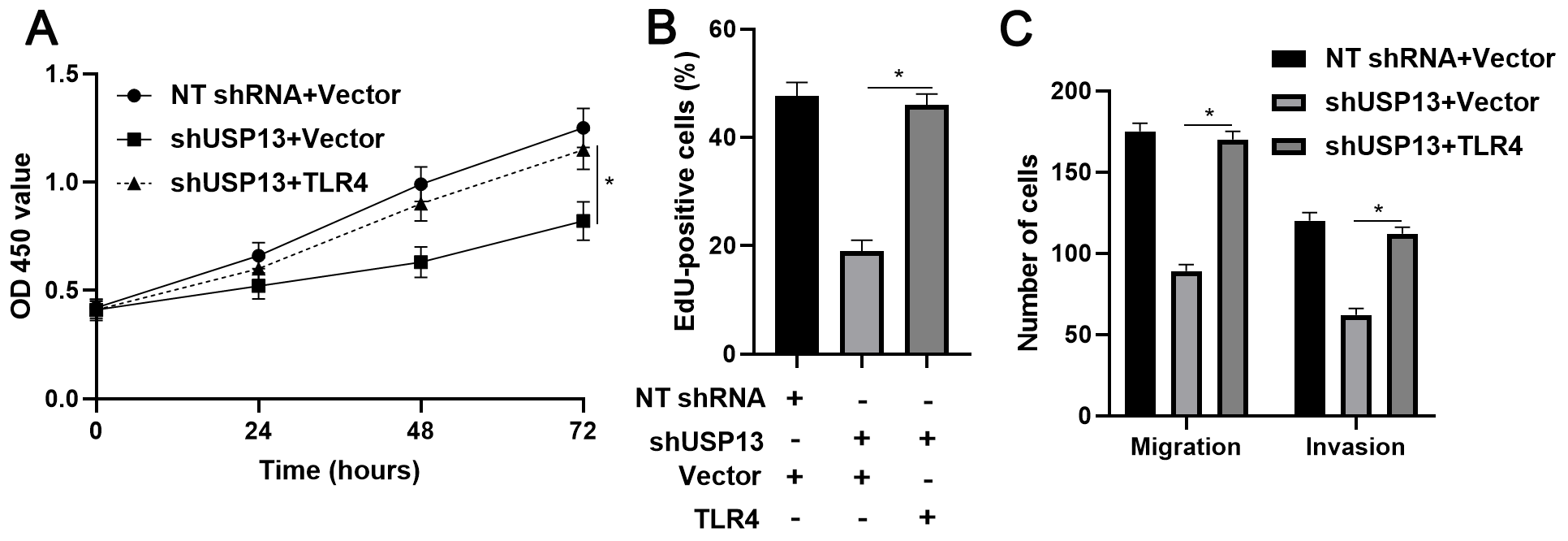

Supplement: Supplementary Figure 5 — TLR4 restoration reverses USP13 knockdown-induced effects on Huh7 cells. (A) CCK-8, (B) EdU, and (C) transwell assays were performed to assess the proliferation, migration, and invasion of Huh7 cells after transfecting with corresponding vectors. ∗P < 0.05. [file Image_5.TIF]
