# Supplementary material for: Hypoxia-Inducible Ubiquitin Specific Peptidase 13 Contributes to Tumor Growth and Metastasis via Enhancing the Toll-Like Receptor 4/Myeloid Differentiation Primary Response Gene 88/Nuclear Factor-κB Pathway in Hepatocellular Carcinoma
Source: Front Cell Dev Biol. 2020 Oct 19;8:587389. doi: 10.3389/fcell.2020.587389 (PMC7604352; doi:10.3389/fcell.2020.587389)
Supplement: Supplementary file 6 [file Table_1.DOCX]

**Supplementary Table 1** **The differently expressed genes in Hep3B cells under hypoxia compared to normoxia**

| **Gene** | **P-value** | **FDR** | **Fold change** |
| --- | --- | --- | --- |
| BNIP3 | 0.000156959 | 0.005707546 | 8.8845108 |
| GLUT1 | 0.001625273 | 0.015806138 | 2.859151 |
| CA9 | 0.000842376 | 0.011312339 | 2.4555879 |
| PDK1 | 0.000430161 | 0.008177802 | 6.0179259 |
| LOXL2 | 0.000463037 | 0.008471111 | 3.8281676 |
| ANGPTL4 | 0.002251054 | 0.019053964 | 6.1695497 |
| PGK1 | 0.005085153 | 0.030014764 | 3.6155171 |
| PLOD1 | 0.000632947 | 0.009801999 | 3.5783099 |
| P4HA1 | 0.00082263 | 0.011230358 | 6.5723144 |
| P4HA2 | 0.001006396 | 0.012454973 | 3.7229072 |
| USP13 | 0.000630194 | 0.009801999 | 6.1790227 |
| TLR4 | 0.007163642 | 0.037154286 | 1.3389295 |
